# Highly accelerated intracranial time‐of‐flight magnetic resonance angiography using wave‐encoding

**DOI:** 10.1002/mrm.29647

**Published:** 2023-04-03

**Authors:** Yang Ji, Wenchuan Wu, Matthijs H. S. de Buck, Thomas Okell, Peter Jezzard

**Affiliations:** ^1^ Wellcome Centre for Integrative Neuroimaging, FMRIB Division, Nuffield Department of Clinical Neurosciences University of Oxford Oxford UK

**Keywords:** 3D time‐of‐flight, compressed sensing, CS‐wave, MR angiography, wave‐CAIPI

## Abstract

**Purpose:**

To develop an accelerated 3D intracranial time‐of‐flight (TOF) magnetic resonance angiography (MRA) sequence with wave‐encoding (referred to as 3D wave‐TOF) and to evaluate two variants: wave–controlled aliasing in parallel imaging (CAIPI) and compressed‐sensing wave (CS‐wave).

**Methods:**

A wave‐TOF sequence was implemented on a 3 T clinical scanner. Wave‐encoded and Cartesian k‐space datasets from six healthy volunteers were retrospectively and prospectively undersampled with 2D‐CAIPI sampling and variable‐density Poisson disk sampling. 2D‐CAIPI, wave‐CAIPI, standard CS, and CS‐wave schemes were compared at various acceleration factors. Flow‐related artifacts in wave‐TOF were investigated, and a set of practicable wave parameters was developed. Quantitative analysis of wave‐TOF and traditional Cartesian TOF MRA was performed by comparing the contrast‐to‐background ratio between the vessel and background tissue in source images, and the structural similarity index measure (SSIM) between the maximum intensity projection images from accelerated acquisitions and their respective fully sampled references.

**Results:**

Flow‐related artifacts caused by the wave‐encoding gradients in wave‐TOF were eliminated by properly chosen parameters. Images from wave‐CAIPI and CS‐wave acquisitions had a higher SNR and better‐preserved contrast than traditional parallel imaging (PI) and CS methods. Maximum intensity projection images from wave‐CAIPI and CS‐wave acquisitions had a cleaner background, with vessels that were better depicted. Quantitative analyses indicated that wave‐CAIPI had the highest contrast‐to‐background ratio, SSIM, and vessel‐masked SSIM among the sampling schemes studied, followed by the CS‐wave acquisition.

**Conclusion:**

3D wave‐TOF improves the capability of accelerated MRA and provides better image quality at higher acceleration factors compared to traditional PI‐ or CS‐accelerated TOF, suggesting the potential use of wave‐TOF in cerebrovascular disease.

## Abstract 2

Click here for author‐reader discussions

## 1 INTRODUCTION

Time‐of‐flight (TOF) is a widely used non‐contrast–enhanced magnetic resonance angiography (MRA) technique that utilizes the magnetization difference between unsaturated spins of inflowing blood and saturated stationary spins to enhance blood vessels. TOF MRA provides excellent depiction of the arterial vasculature and is routinely used in clinical brain exams to evaluate cerebrovascular diseases such as stenosis and aneurysm.^1^, ^2^ TOF sequences can be deployed either in 2D or 3D configurations, which have specific technical implementations that make them more suitable for particular clinical applications.^3^, ^4^ The 3D sequences have a generally higher SNR that can be used to acquire a volume with high spatial resolution in all three spatial directions,^5^ making them a preferable choice for depicting smaller vascular structures and, in turn, improving the diagnostic accuracy of detecting cerebrovascular disease. With multiple overlapping thin 3D slab acquisitions,^6^ it is possible to combine the contrast advantage of 2D acquisitions with the higher SNR of 3D acquisitions to further reduce blood signal saturation while maintaining SNR. However, the main disadvantage of 3D‐TOF MRA is the long acquisition time when acquiring multiple slabs and covering a large FOV, especially for high spatial resolution imaging.^7^ A long scan time also potentially increases the risk of motion artifacts, which may reduce image quality and impair radiological confidence in image diagnosis.^8^ Parallel imaging (PI) approaches, such as GRAPPA^9^ and SENSE,^10^ have been used to reduce scan time by undersampling k‐space. However, the PI acceleration factor used for TOF is still limited by coil geometry, and higher acceleration factors may cause a rapid increase in noise and aliasing artifacts. Consequently, an acceleration factor >3 is not commonly used in clinical routine due to unreliable image quality. Compressed sensing (CS) MRI is another advanced image reconstruction technique^11^ that has been intensively investigated in TOF MRA because of its great potential to reduce scan time.^12^, ^13^, ^14^ CS exploits the incoherently undersampled k‐space data and sparsity of the TOF image in an appropriate transform domain to achieve a very high acceleration factor while maintaining good image quality, which is promising for clinical applications. However, the slow computation speed and the need for parameter tuning are major drawbacks of CS‐MRI.^15^

Wave‐controlled aliasing in parallel imaging (wave‐CAIPI) is an emerging parallel imaging technique,^16^, ^17^, ^18^, ^19^ which has been demonstrated to achieve highly accelerated 3D volume imaging with low artifact and SNR penalties and has been used in various applications.^20^, ^21^, ^22^, ^23^, ^24^ In addition to employing a 2D‐CAIPI PI technique,^25^ wave‐CAIPI primarily applies sinusoidal gradients with a π/2 phase shift along phase and partition encoding directions during the readout of each encoding line, resulting in corkscrew k‐space trajectories that are well suited for PI. Compared with rectangular undersampling in a Cartesian sampling trajectory, this non‐Cartesian undersampling provides a more efficient k‐space coverage. The aliasing pattern resulting from a wave‐encoding is not only spread in the phase and partition encoding directions but also in the frequency encoding direction. As a result, coil sensitivity variations along three directions can be exploited more efficiently, leading to a further improvement in PI reconstruction conditions and, therefore, a better image quality. Although the sampling trajectory is non‐Cartesian, a standard SENSE framework for Cartesian k‐space data can still be used for the reconstruction of wave‐CAIPI, in which the point spread function (PSF) characterizing the encoding effect of the wave gradients is incorporated into the encoding matrix of a generalized SENSE mode in the hybrid *k*_x_‐*y*‐*z* space.

In this work, we evaluated the feasibility of combining 3D‐TOF MRA with the wave‐CAIPI technique to accelerate imaging speed and to improve the imaging quality of the cerebral vasculature. The first‐order moment of the sinusoidal gradients also introduces an additional modulated phase to moving spins,^26^ which can lead to flow‐related ghosting artifacts for spins with a high flow velocity. In order to suppress such flow‐related artifacts, while maintaining good image quality, the parameters of the wave gradients must be carefully chosen. Once established, 3D wave‐TOF MRA was compared to conventional intracranial TOF MRA at different acceleration factors. Because previous studies have demonstrated the feasibility of intracranial TOF MRA with CS‐MRI to reduce scan time, CS was also combined with wave‐encoding (dubbed as *CS‐wave*), and the performance of both wave‐CAIPI and CS‐wave TOF was evaluated.

## 2 METHODS

### 2.1 Sequence implementation and data acquisition

**FIGURE 1:**
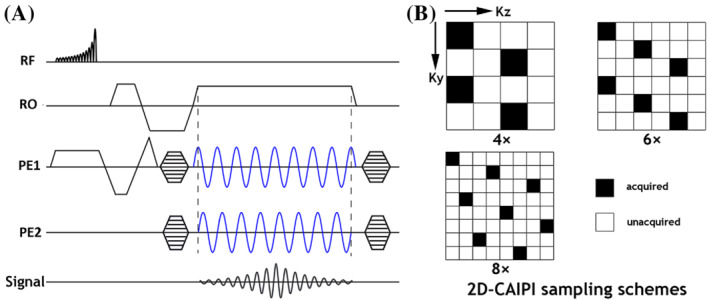
(A) Pulse sequence diagram of wave‐encoded TOF (wave‐TOF) MRA. Note that an asymmetric TONE pulse is used for excitation. Two wave‐encoding gradients with π/2 phase difference are applied during the readout period, rendering a corkscrew k‐space trajectory. (B) The 2D‐CAIPI sampling masks with acceleration rates of *R* = 4, 6, and 8, which generate controlled aliasing by shifting sampling positions in the 2D phase encoding scheme with respect to each other. CAIPI, controlled aliasing in parallel imaging; TOF, time of flight.

A schematic diagram of the proposed 3D wave‐TOF sequence is shown in Figure 1A. The sequence was implemented based on a standard 3D‐TOF sequence, with additional sinusoidal gradients applied in both phase and partition encoding directions during the readout period. The gradients were shifted relative to one another by a phase of π/2, resulting in a corkscrew‐shaped readout trajectory. To acquire the k‐space data more efficiently, a 2D‐CAIPI sampling pattern in k_y_‐k_z_ space was adopted. Figure 1B shows three 2D‐CAIPI sampling patterns with acceleration factors of 4, 6, and 8, respectively. These sampling patterns have the lowest mean g‐factor among alternative patterns with the same acceleration factor for traditional PI^25^ and were evaluated for the CAIPI variant of wave‐TOF.

All MRI experiments were performed on a 3 T scanner (Magnetom Prisma, Siemens Healthineers, Erlangen, Germany) equipped with a gradient coil with a maximum strength of 80 mT/m and a maximum slew rate of 200 T/m/s, and a 32‐channel head receive coil. All in vivo data were acquired from six healthy young males (age range 23–34 years) and were approved under a technical development protocol by the local institutional review board. Four slabs were fully sampled in each subject with a slab overlap factor of 13.64% to cover the brain using the proposed 3D wave‐TOF MRA sequences, which were retrospectively undersampled in the image reconstruction. Additional prospectively undersampled datasets were acquired using the proposed 3D wave‐TOF and conventional Cartesian 3D‐TOF MRA. The common parameters used in the TOF sequences studied were as follows: matrix size per slab = 256 × 256 × 44 (note: 44 represents the usable partitions per slab), resolution = 0.8 mm^3^ × 0.8 mm^3^ × 0.6 mm^3^, FOV = 200 mm^3^ × 200 mm^3^ × 106 mm^3^ (4 slabs), TR/TE = 14/3.5 ms (asymmetric echo used to reduce TE), flip angle = 20°, and bandwidth = 121 Hz/pixel. For the wave‐TOF scans, an oversampling factor of 6 was used in the frequency encoding direction to ensure that the wave images do not suffer from circular convolution and the voxel spreading does not cause pixel wraparound along the readout direction. To avoid flow‐related artifacts caused by the wave‐encoding gradients in the wave‐TOF sequence, several sets of the wave‐encoding parameters were evaluated, and a cycle number (*N*_cyc_) of 15 and an absolute wave amplitude (G_max_) of 10 mT/m were finally chosen for the wave‐TOF MRA protocol. The corresponding spread‐out range of PSF calculated using Equation 4 in Ref. 27 was approximately 296 mm. The scan time was 6:00 min for a fully sampled acquisition of each slab. In addition, a separate 3D‐FLASH reference scan that acquired the central 30 × 30 region of k_y_‐k_z_‐space was performed for estimation of sensitivity maps using ESPIRiT.^28^ Imperfections in the gradient system due to hardware‐related timing delays and eddy current‐induced waveform distortions can cause deviations between the real wave gradients and the nominal ones. These discrepancies lead to inaccuracy in the k‐space trajectory, consequently resulting in severe artifacts. To measure the real k‐space trajectory of the wave gradients, a fast trajectory calibration measurement was performed using the method proposed by Duyn et al.^29^ including four projection scans in the phase and slice directions performed with and without the application of the sinusoidal gradients. Because the real trajectory depends only on the specific gradient system, and not on the subject being scanned, the trajectory calibration process only needs to be done once and will be valid for any subsequent acquisition of the same pulse sequence protocol.

### 2.2 Reconstruction

Images were reconstructed offline from the raw k‐space data with an in‐house program coded in MatLab R2021b (MathWorks, Natick, MA). Coil sensitivity maps were estimated with the ESPIRiT method^28^ via the Berkeley Advanced Reconstruction Toolbox (BART)^30^ from the 3D‐FLASH reference scan. Considering the long scan time and possibility of motion of the subjects, fully sampled datasets for the whole brain using conventional TOF MRI were not acquired. Instead, synthesized fully sampled Cartesian k‐space datasets were generated by deconvolving the fully sampled wave‐encoded datasets in *k*_x_‐*y*‐*z* space using the measured PSF.^16^ The fully sampled synthetic Cartesian dataset then serves as the reference data. Next, the fully sampled wave‐encoded data and the synthesized fully sampled Cartesian k‐space data were retrospectively undersampled using the 2D‐CAIPI undersampling mask patterns illustrated in Figure 1B to achieve total acceleration factors (R) of 4, 6, and 8. Both retrospectively and prospectively undersampled wave‐CAIPI data were reconstructed by solving the following minimization problem with an iterative method (LSQR) in a Cartesian framework:

(1) $$\min_{s}{\left\|M\cdot F_{z}\cdot F_{y}\cdot \mathrm{PSF}\left(k_{x},y,z\right)\cdot F_{x}\cdot C\cdot s-k\right\|}_{2}^{2}\;,$$

where *M* is the mask of the 2D‐CAIPI sampling pattern; *F*_*x*_, *F*_*y*_, and *F*_*z*_ are the Fourier transform operators along the *x*, *y*, and *z* axes in image space, respectively; $\mathrm{PSF}\left(k_{x},y,z\right)$ is the point spread function of the wave‐encoding gradients; *C* is the estimated coil sensitivity; *s* is the unknown image to be reconstructed; and *k* is the undersampled wave‐encoded k‐space data. The tolerance and maximum number of iterations for the LSQR were set to 1e‐3 and 200, respectively. Similarly, the undersampled Cartesian data were reconstructed using the same method but without the PSF term.

We also investigated the use of the CS reconstruction method on wave‐encoded data as a way of accelerating the acquisition of TOF MRA. Instead of the 2D‐CAIPI sampling masks, the CS‐wave method used sampling masks with the same acceleration factors that were generated using pseudorandom variable‐density Poisson disks. The desired images were then recovered from the undersampled CS‐wave dataset by solving the following optimization problem with a fast nonlinear conjugate gradient (NLCG)–based algorithm^31^, ^32^ with L1‐regularization in the gradient and wavelet domains:

(2) $$\begin{array}{ll} & \min_{s}{\left\|M\cdot F_{z}\cdot F_{y}\cdot \mathrm{PSF}\left(k_{x},y,z\right)\cdot F_{x}\cdot C\cdot s-k\right\|}_{2}^{2}\\ & \;+\gamma_{G}{\left\|G\cdot s\right\|}_{1}+\gamma_{W}{\left\|W\cdot s\right\|}_{1}, \end{array}$$

where G and W denote the gradient and Haar wavelet transforms of s, respectively. $\gamma_{G}$ and $\gamma_{W}$ are the regularization weights that govern the tradeoff between the data consistency and the sparsity regularization terms, and both were set to 3e‐4. The tolerance and maximum number of iterations for the L1 penalized NLCG reconstruction were set to 3e‐3 and 100, respectively. Reconstruction of the sub‐sampled Cartesian dataset was performed with the CS algorithm, but without the PSF term, at the same sparsity regularization condition. The MatLab R2021b (MathWorks) code used for the reconstructions performed in this study is available at https://github.com/yangji6/WaveTOF.

### 2.3 Image analysis

G‐factor maps were used to compare the quality of source images reconstructed from wave‐CAIPI and 2D‐CAIPI with different acceleration factors, which were calculated using the Monte Carlo method proposed by Robson et al.^33^ with 100 pseudo multiple replicas. The maximum and mean g‐factor (g_max_ and g_mean_) were evaluated in the region of interest. Contrast‐to‐background ratio (CBR) between cerebral arteries and background tissue^12^ in source images was measured to evaluate the visibility of vessels over the background with various acceleration approaches. The CBR was defined as the ratio of the signal difference between the vessel and the background tissue versus the signal variation of the background tissue signal with the following formula:

(3) $$\mathrm{CBR}=\left({\text{Mean}}_{\text{vessel}}-{\text{Mean}}_{\text{background}}\right)/{\mathrm{SD}}_{\text{background}},$$

where ${\text{Mean}}_{\text{vessel}}$ is the averaged signal intensity of the vessels, and ${\text{Mean}}_{\text{background}}$ and ${\mathrm{SD}}_{\text{background}}$ are the averaged signal intensity and SD of the entire static background tissue in the source images, respectively. Maximum intensity projection (MIP) images were generated from the source images of the TOF MRA. As previous work reported that the vessel‐masked structural similarity index measure (SSIM) well correlates with visual assessment by radiologists when evaluating the reconstructions from retrospectively undersampled TOF MRA dataset,^13^, ^34^ we adopted the vessel‐masked SSIM in addition to a standard SSIM in this study to assess the accuracy of the reconstruction from retrospectively undersampling. Additionally, CBR, SSIM, and vessel‐masked SSIM metrics were compared between different accelerated sampling schemes using a two‐tailed paired *t* test with Bonferroni correction, and adjusted *P* values less than 0.05 were considered statistically significant (*N* = 6).

## 3 RESULTS

**FIGURE 2:**
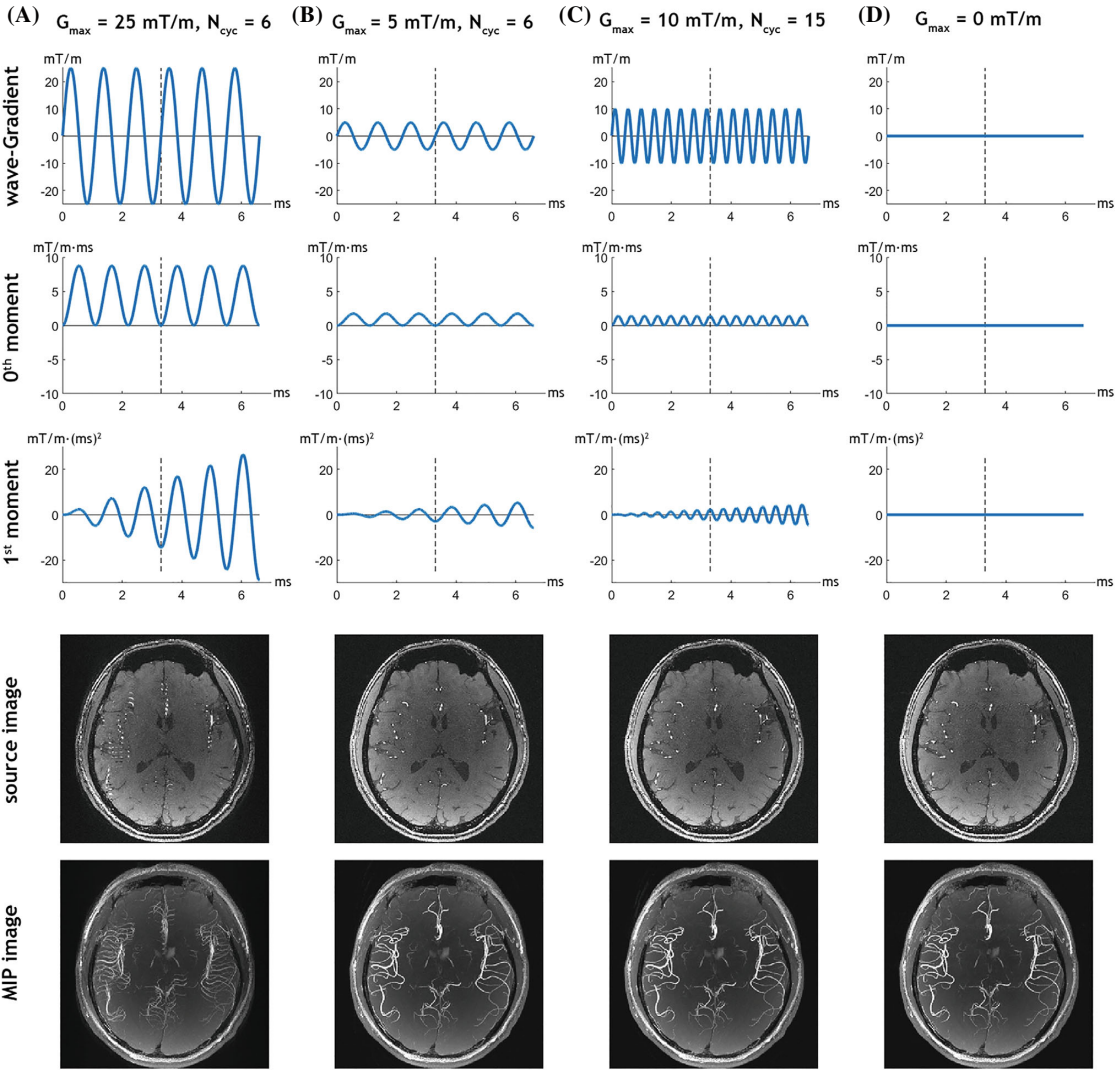
The waveforms of the wave‐encoding gradients (first row) and the corresponding zeroth‐ (second row) and first‐ (third row) order moments during the readout period, as well as a source image of a representative slice (fourth row) and a MIP image of a slab (bottom row) obtained from wave‐TOF acquisitions with different *G*_max_ and *N*_cyc_ parameters: (A) *G*_max_ = 25 mT/m, *N*_cyc_ = 6, (B) *G*_max_ = 5 mT/m, *N*_cyc_ = 6, (C) *G*_max_ = 10 mT/m, *N*_cyc_ = 15, and (D) *G*_max_ = 0 mT/m (reference: Cartesian sampling). *G*_max_, absolute wave amplitude; *N*_cyc_, cycle number; MIP, maximum intensity projection.

To demonstrate the flow‐related artifact caused by the wave‐encoding gradients in wave‐TOF, and to obtain a flow artifact‐free imaging protocol, three representative sets of the wave‐encoding parameters were evaluated. Figure 2 shows the evolutions of the zeroth‐ and first‐order moment of the wave‐encoding gradient with different values of *N*_cyc_ and *G*_max_ during the readout period, along with a representative source image and a MIP image of a slab obtained by the wave‐TOF sequence. Figure 2A shows the results with *N*_cyc_ = 6 and *G*_max_ = 25 mT/m. Whereas the zeroth‐order moment is a sinusoidal oscillation with a constant amplitude, the first‐order moment is an oscillation with an amplitude that increases with time. The oscillation of the first‐order moment causes a periodic modulation of fluid signal phase that can potentially introduce flow‐related ghosting artifacts. The images in Figure 2A show noticeable ghosting of the vessel signal replicated along the frequency encoding direction due to the phase modulation from the first‐order moment. With a decrease of the wave amplitude (*G*_max_ = 5 mT/m and *N*_cyc_ = 6) in Figure 2B or an increase of the cycle number (*G*_max_ = 10 mT/m and *N*_cyc_ = 15) in Figure 2C, the amplitude of the oscillation in the first‐order moment is decreased and the flow artifacts in the source image and MIP image in both Figure 2B,C are negligible. These results indicate that the flow‐related artifacts caused by the wave‐encoding gradient can be significantly suppressed by decreasing the oscillation amplitude of the first‐order gradient moment.

**FIGURE 3:**
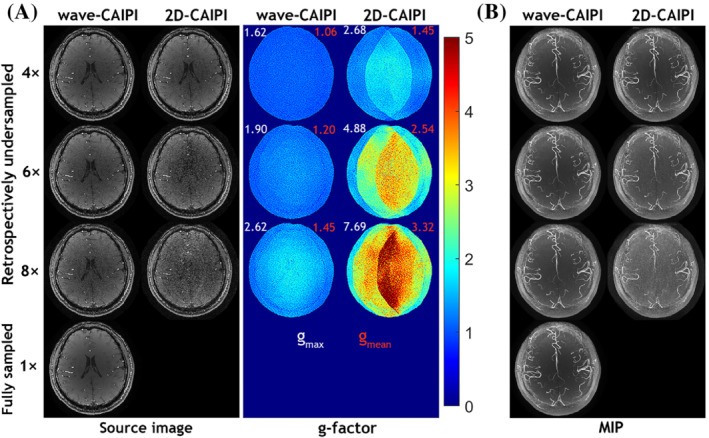
Comparison of TOF MRA with wave‐CAIPI and regular 2D‐CAIPI at acceleration factors of 4, 6, and 8 with the corresponding sampling schemes demonstrated in Figure 1B. (A) Reconstructed source images of a representative slice within one slab from retrospectively undersampled wave‐CAIPI, regular 2D‐CAIPI, and fully sampled acquisitions as well as the respective g‐factor maps for each source image. (B) MIP images of one slab from retrospectively undersampled wave‐CAIPI, regular 2D‐CAIPI, and fully sampled acquisitions

To visualize the performance of the wave‐CAIPI technique in TOF MRA, comparisons between the images from accelerated wave‐CAIPI TOF and conventional TOF are shown in Figure 3. Figure 3A shows the reconstructed source images of a representative slice from retrospectively undersampled acquisitions with acceleration factors of 4, 6, and 8 and the fully sampled source image that serves as the ground‐truth reference, as well as the respective g‐factor maps for each source image. As expected, the source images from wave‐CAIPI have a much lower and more homogeneous g‐factor than conventional CAIPI TOF. Figure 3B compares the MIP images from an accelerated wave‐CAIPI acquisition versus a conventional CAIPI imaging scheme. All MIP images from the wave‐CAIPI acquisition with different acceleration factors have a relatively clean background and the vessels are well depicted, whereas the background in the MIPs from a conventional CAIPI acquisition is much noisier, especially at *R* = 8.

**FIGURE 4:**
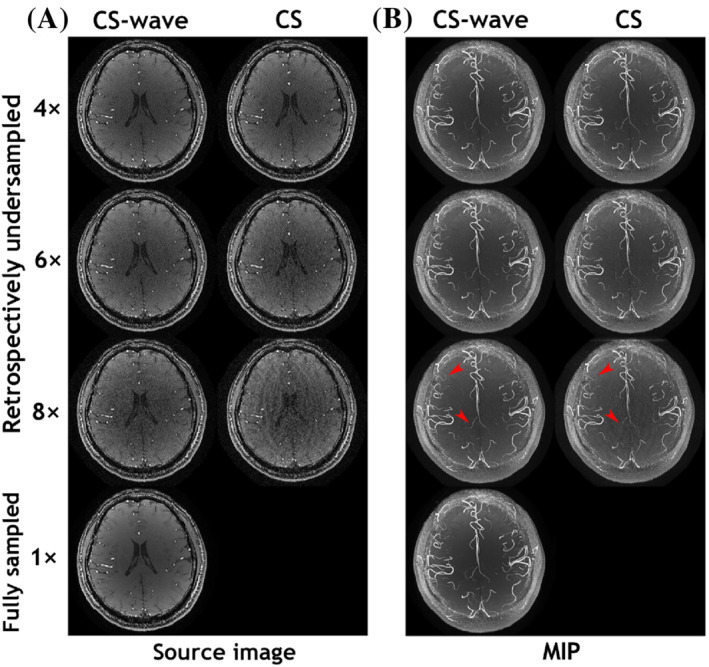
Comparison of TOF MRA with CS‐wave and regular CS at acceleration factors of 4, 6, and 8 with the corresponding variable‐density Poisson‐disc sampling patterns. (A) Reconstructed source images of a representative slice within one slab from retrospectively undersampled CS‐wave, regular CS, and fully sampled acquisitions. (B) MIP images of one slab from retrospectively undersampled CS‐wave, regular CS, and fully sampled acquisitions. The red arrows indicate locations with improved small vessel visibility when using CS‐wave compared to conventional CS at *R* = 8. CS, compressed‐sensing.

As depicted in Figure 3, the wave‐CAIPI technique demonstrates its superiority for vessel contrast, and the robustness of wave‐CAIPI to noise is an obvious advantage compared to a conventional imaging scheme. Next, we exploit the integration of wave‐encoding, random sampling, and CS reconstruction with a sparsity prior (CS‐wave) to accelerate the acquisition and improve the image quality of intracranial TOF MRA. Figure 4 compares the results of retrospectively accelerated intracranial TOF MRA with CS‐wave and traditional CS methods. The source images from CS‐wave retain a good image quality with relatively high SNR even at an undersampling factor of 8. In comparison, aliasing artifacts can be easily observed in the conventional CS image but not in the CS‐wave result. Figure 4B compares the MIP images from the CS‐wave and conventional CS imaging schemes. The MIP images from both methods provide similar vascular representation and do not exhibit a noisy background like in Figure 3B. However, the vessels in the MIP images from the CS‐wave method have a higher standard SSIM and vessel‐masked SSIM than those from conventional CS, particularly at high acceleration factors (i.e., standard SSIM: 0.83 vs. 0.70; vessel‐masked SSIM: 0.98 vs. 0.94, at *R* = 8). Consequently, CS‐wave provides a higher CBR between vessel and background tissue than conventional CS. The red arrows show that conventional CS at *R* = 8 provided a reduced visualization of small vessels compared to CS‐wave due to the decreased CBR. The CS‐wave technique demonstrates its advantage with vessel contrast and continuity compared with the regular CS technique.

**FIGURE 5:**
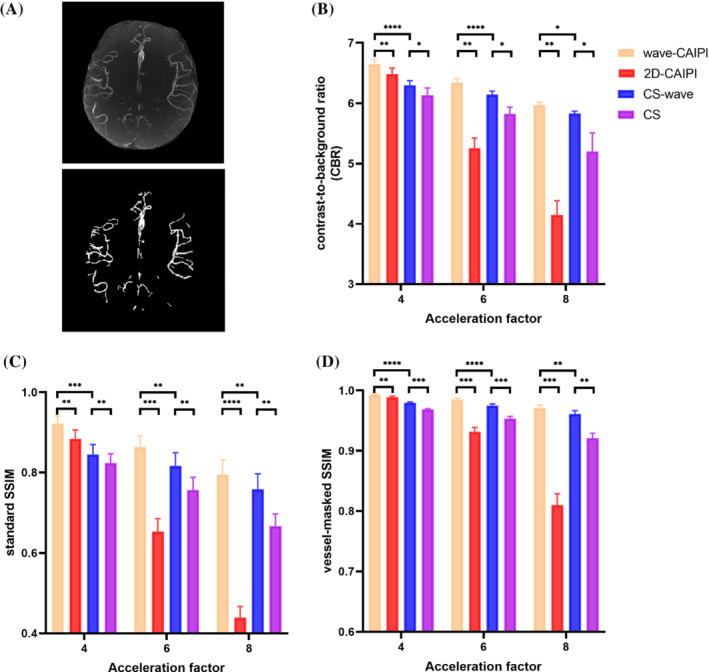
(A) MIP image of a representative slab and the corresponding vessel mask that was used for the calculation of the CBR and vessel‐masked SSIM. (B) CBR between vessels and static background tissue at different acceleration factors for TOF MRA with different sampling schemes. (C) Standard SSIM between the MIP images obtained from the undersampled and fully sampled reconstructions. (D) Vessel‐masked SSIM between the MIP images with vessel masks obtained from the undersampled and fully sampled reconstructions. (*P* values are indicated by stars: * *P* < 0.05, ** *P* < 0.01, *** *P* < 0.001, ****P < 0.0001). CBR, contrast‐to‐background ratio; SSIM, structural similarity index measure.

Figure 5A shows an example of a MIP image and the corresponding vessel mask, which was used for calculating the CBR and the vessel‐masked SSIM. Figure 5B shows the quantitative analyses of the CBR between vessels and static background tissue, which were generally consistent with the visual observation in Figures 3 and 4. As shown in Figure 5B, wave‐CAIPI performed best when taking CBR as an evaluation criterion, with the highest CBR among the four sampling schemes at different acceleration factors. With an increase in acceleration factor, the CBR of the 2D‐CAIPI and conventional CS decreases noticeably (e.g., R4 to R8, 2D‐CAIPI: 6.48 ± 0.09 to 4.15 ± 0.20, *P* < 0.001; CS: 6.11 ± 0.06 to 5.12 ± 0.20, *P* < 0.01), whereas the CBR of the wave‐CAIPI and CS‐wave techniques decreased only slightly (e.g., R4 to R8, wave‐CAIPI: 6.65 ± 0.07 to 5.97 ± 0.04, *P* < 0.0001; CS‐wave: 6.30 ± 0.07 to 5.83 ± 0.03, *P* < 0.001). Moreover, the CBR of the CS‐wave scheme is significantly lower than that of the wave‐CAIPI scheme but significantly higher than that of the conventional CS scheme at all acceleration factors (e.g., R6, wave‐CAIPI: 6.34 ± 0.05; CS‐wave: 6.14 ± 0.05; CS: 5.82 ± 0.10). Figure 5C,D show the standard SSIM and vessel‐masked SSIM between the MIP images obtained from the undersampled reconstruction and the reference dataset from the fully sampled reconstruction without and with vessel masking, respectively. The trends of the standard SSIM and vessel‐masked SSIM are generally consistent with that of the CBR results in Figure 5A. The vessel‐masked SSIMs of the wave‐CAIPI and CS‐wave schemes decrease slightly with increasing acceleration factor (e.g., R4 to R8, wave‐CAIPI: 0.993 ± 0.001 to 0.971 ± 0.004, *P* < 0.01; CS‐wave: 0.980 ± 0.001 to 0.961 ± 0.005, *P* < 0.01), suggesting that the vessel signal intensity can be adequately retained at a high acceleration factor using wave‐encoding. However, the standard SSIM values decrease more rapidly with increasing acceleration factor than the vessel‐masked SSIM values (e.g., R4 to R8, wave‐CAIPI: 0.923 ± 0.016 to 0.795 ± 0.031, *P* < 0.001; CS‐wave: 0.845 ± 0.022 to 0.758 ± 0.033, *P* < 0.01), indicating that the noise in the static background tissue was amplified under these conditions. The qualitative metrics reported above are also summarized in Table S1.

**FIGURE 6:**
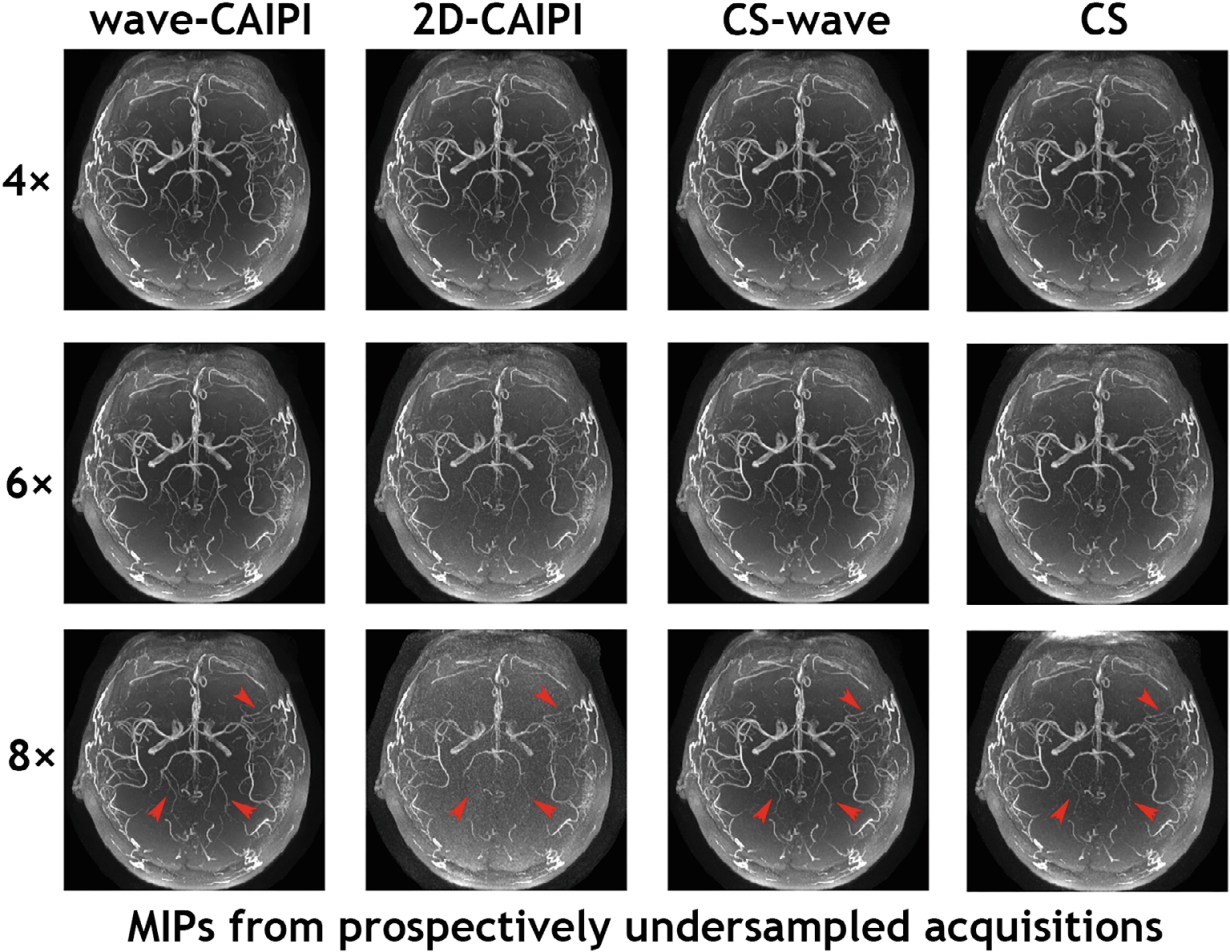
Whole‐brain axial MIP images obtained from all four slabs with various prospectively undersampled schemes: wave‐CAIPI, 2D‐CAIPI, CS‐wave, and conventional CS at acceleration factors of 4, 6, and 8, respectively

Figure 6 shows an example of the whole‐brain axial MIP images obtained from all four slabs with various prospectively undersampled schemes: wave‐CAIPI, 2D‐CAIPI, CS‐wave, and conventional CS at acceleration factors of 4, 6, and 8. Visually, 2D‐CAIPI performs the worst at high acceleration factors (e.g., 6 and 8) with an obvious noisy background when compared with other sampling schemes with the same acceleration factor. However, the vessel structures are much better preserved in the MIP images from wave‐CAIPI and CS‐wave. A vascular discontinuity can be observed in MIP images from conventional CS at an acceleration factor of 8, whereas the continuity of vessels at the same location is preserved in the MIP images from the wave‐CAIPI and CS‐wave techniques, consistent with the comparison of the quantitative metrics shown in Figure 5.

## 4 DISCUSSION

In this study, we developed a wave‐TOF sequence to accelerate acquisition and to improve image quality. TOF MRA is based on flow‐related enhancement, but flow‐related artifacts can also be encountered when additional gradients are applied during the readout (Figure 2). The wave‐encoding gradients not only introduce a wave‐modulated zeroth‐order moment but also a wave‐modulated first‐order moment.^26^ The additional phase shift induced by the zeroth‐order moment can be addressed in *k*_x_‐*y*‐*z* space using the measured PSF during the reconstruction stage. However, phase shifts induced by first‐ or higher‐order moments (the source of flow‐related ghosting artifacts) cannot easily be corrected. The underlying theory of such artifacts is very similar to that of pulsatile flow artifacts or periodic motion artifacts,^35^, ^36^, ^37^ where the signal ρ(*x*, *y*) of blood located at (*x*, *y*) in conventional TOF becomes a periodic function of time due to periodic flow or motion. This causes inconsistency between phase encoding lines, leading to ghosting artifacts along the phase encoding direction. In wave‐TOF, the desired signal ρ(*x*, *y*) is modulated by the phase variations introduced from periodic wave‐encoding gradients. Because the signal modulation occurs among sampling points within each phase encoding line, the ghosting appears in the frequency encoding direction rather than the phase encoding direction. We have found that these flow artifacts are noticeable in wave‐TOF images when the wave‐encoding gradients have a large first‐order moment, which is associated with a large phase shift for moving spins. The flow artifacts can be successfully eliminated by increasing the number of cycles or decreasing the maximum amplitude of the wave‐encoding gradients concurrently, and hence decreasing the phase shift. The phase shift due to the wave‐encoding gradient can also be minimized by reducing the duration between the start time of ADC acquisition and the TE,^37^ which can be achieved by using an asymmetric echo or a higher bandwidth to further suppress the ghosting.

To maximize the capability of the wave‐TOF schemes, the gradient waveforms (number of oscillations and wave amplitudes) should be optimized. Previous studies have shown that a larger wave amplitude leads to a greater voxel spreading effect and consequently to a smaller g‐factor penalty in the final reconstructed images, whereas the number of cycles minimally affects the g‐factor.^27^ However, the parameters best suited for suppressing vessel ghosting in wave‐TOF can limit its capability in reducing the g‐factor because a smaller wave amplitude and a larger number of oscillations are beneficial for reducing the first‐order moment and thereby the ghosting artifacts. When considering the slew rate limit of the gradients and peripheral nerve stimulation, the number of cycles, wave amplitude, and bandwidth should be carefully chosen to maximize the performance of the wave‐CAIPI technique while avoiding ghosting artifacts. In this study, we only demonstrate one example set of feasible parameters that yields ghosting‐free wave‐TOF. The best tradeoff between the number of wave cycles, bandwidth, and the wave amplitude for ghosting‐free wave‐TOF will be the subject of future study.

The PI reconstruction process can be characterized as a linear inverse problem that recovers an unfolded image from the undersampled k‐space dataset.^38^ The recovered images can be obtained with minimal amplification of noise at a low acceleration factor, which has a well‐conditioned system matrix. The matrix becomes ill‐conditioned when the acceleration factor is high, where small noise perturbations in the measured data can lead to large noise amplification. The wave‐TOF approach improves the conditioning of the encoding matrix by efficiently exploiting coil sensitivity variations in all three dimensions and results in relatively low noise amplification even at a high acceleration factor. In this study, we compared a wave‐CAIPI TOF MRA scheme with a 2D‐CAIPI TOF MRA scheme. We further combined CS with wave‐encoding for TOF MRA and evaluated the performance of CS‐wave. Interestingly, wave‐CAIPI performed better than CS‐wave regarding both the CBR and the SSIM metric. This result is contrary to previous similar studies,^32^, ^39^ in which CS‐wave outperformed wave‐CAIPI on normalized RMS error. This may be caused by several factors. Firstly, the different focus of TOF MRA and regular MRI may lead to a different quantitative result. The spins of static tissue are saturated in TOF MRA with the goal of suppressing static signal as much as possible, whereas regular MRI mainly focuses on the static tissues and aims to image them at a high SNR, thereby achieving different image contrasts. Secondly, the CS‐wave reconstruction used here may not be optimized because the performance of the CS‐based methods strongly depends on the parameter tuning and the undersampling patterns, which may affect the quantitative indices. It is worth mentioning that the CBR metric was adopted rather than the contrast‐to‐noise ratio (CNR) for evaluating the performance of the proposed method. Although the calculation of CBR does not need the true noise level of the source TOF images, CBR can be considered an estimator of the CNR to assess how noisy the images are. It also should be noted that the fully sampled Cartesian dataset in the retrospectively undersampled study was derived from the wave‐encoded non‐Cartesian dataset rather than directly acquired with a Cartesian sampling trajectory. This process ensures the two images compared in any SSIM measurement are spatially consistent, which avoids interference from minor subject motion in the analysis. However, we also needed to ensure that this process would not bias the comparative analysis. To validate this, both fully sampled Cartesian and wave‐encoded datasets for one slab were acquired. The source images and MIPs from retrospectively undersampled reconstructions with the same acceleration factor for both datasets are almost identical, as shown in Figures S1 and S2, indicating that the performance difference between wave‐encoded sampling and conventional Cartesian PI sampling is mainly attributed to the wave‐encoding technique itself.

Because imperfections in the gradient system can cause the physical waveforms to deviate from the nominal ones, the actual k‐space trajectory of the sinusoidal gradients needs to be measured for accurate image reconstruction. In this study, we performed trajectory measurement using a simple calibration method.^29^ Because the gradient property is relatively stable on modern MRI scanners, the calibration process only needs to be done once and thus will not incur extra scan time in later scans with the same image protocol. However, the calibration measurement needs to be performed for each different set of protocol parameters (e.g., bandwidth, matrix size sinusoidal amplitude/frequency). Additionally, the timing of the ADC relative to the sinusoidal gradients in the trajectory measurement sequence must match exactly with that of the wave‐TOF sequence, because even a small (e.g., 1 μs) timing shift can cause visible artifacts, compromising the scan procedure. Recently, a data‐driven calibration method^40^ has also been proposed, allowing for the auto‐calibration of gradient trajectories for wave‐encoded datasets, thereby avoiding an additional trajectory measurement. Due to the multiple oversampling used in wave‐encoded acquisitions along the frequency encoding direction, the size of the k‐space dataset is many times larger than the traditional TOF MRA. To alleviate the speed and memory constraints that emerge during the reconstruction stage for a wave‐encoded dataset, a coil compression method^41^ can be exploited to compress the dataset along the coil dimension, facilitating fast and efficient computation. In this work, the wave‐TOF data were reconstructed offline using MatLab R2021b (MathWorks) code, which was time‐consuming, even though we parallelized the reconstruction of different slabs. For example, the averaged reconstruction time of one slab with wave‐CAIPI, 2D‐CAIPI, CS‐wave, and standard CS methods at *R* = 8 are 10, 22 min, 1 h 29 min, and 1 h 21 min, respectively. A reconstruction pipeline for wave‐TOF will be prepared using Gadgetron^42^ and the BART^30^ software package in the future to make wave‐TOF MRA available for routine clinical diagnosis. It is also important to note that the NLCG approach used for CS reconstruction in this study may not be the most efficient approach for problems involving the l1‐norm. In such cases, it would be beneficial to consider newer and more efficient approaches like the ADMM or FISTA methods^43^, ^44^ for wave‐CS, especially when translating this method to future clinical use. Although wave‐TOF has demonstrated its superiority in healthy volunteers in this work, the performance and the diagnostic quality in patients with cerebrovascular disorders needs to be further investigated.

## 5 CONCLUSION

In this study, we applied the wave‐encoding approach to accelerated 3D intracranial TOF MRA and evaluated its performance with various acceleration factors. We found that 3D wave‐TOF can significantly improve the capability of PI and achieve better depiction and sharpness of vessels at high acceleration factors compared to traditional PI TOF or CS TOF, facilitating the use of wave‐TOF in clinical routine for screening cerebrovascular diseases.

## FUNDING INFORMATION

This study was supported by the Oxford National Institute for Health and Care Research (NIHR) Biomedical Research Centre (BEC). The Wellcome Centre for Integrative Neuroimaging (WIN) is supported by core funding from the Wellcome Trust (203139/Z/16/Z).

We also thank the Dunhill Medical Trust (DMT) for (P.J., M.H.S.deB.); the Royal Academy of Engineering (RAEng) for (W.W.) grant (RF201819/18/92;); and the grant support from Siemens Healthineers (M.H.S.deB.).

T.O. is supported by a Sir Henry Dale Fellowship jointly funded by the Wellcome Trust and the Royal Society (220204/Z/20/Z).

## CONFLICT OF INTEREST

p.j. is Editor‐in‐Chief of *Magnetic Resonance in Medicine*. In line with COPE guidelines, he recused himself from all involvement in the review process of this paper, which was handled by an Associate Editor. He and the other authors have no access to the identity of the reviewers.

## Supporting information


**FIGURE S1.** Comparison of retrospectively undersampled reconstructions using the 2D‐CAIPI sampling scheme at different acceleration factors for a synthesized Cartesian dataset derived from a fully sampled wave‐encoded dataset and a reference Cartesian dataset acquired using the conventional method.
**FIGURE S2.** Comparison of retrospectively undersampled reconstructions using the CS sampling scheme at different acceleration factors for a synthesized Cartesian dataset derived from a fully sampled wave‐encoded dataset and a reference Cartesian dataset acquired using the conventional method.
**TABLE S1.** Results from the quantitative analyses of the CBR between vessels and static background tissue at different acceleration factors, SSIM and Vessel‐masked SSIM between the MIP images obtained from the undersampled and fully sampled reconstructions (*N* = 6).

## Data Availability

In support of *Magnetic Resonance in Medicine*'s reproducible research goal, the MatLab code used for the reconstructions performed in this study is available at https://github.com/yangji6/WaveTOF. A dataset used for testing the reconstruction code are available from the authors, upon reasonable request.

## References

[1] Degnan A , Gallagher G , Teng Z , Lu J , Liu Q , Gillard J . MR angiography and imaging for the evaluation of middle cerebral artery atherosclerotic disease. Am J Neuroradiol. 2012;33:1427‐1435. 21940802 10.3174/ajnr.A2697 PMC7966534

[2] Deutschmann H , Augustin M , Simbrunner J , et al. Diagnostic accuracy of 3D time‐of‐flight MR angiography compared with digital subtraction angiography for follow‐up of coiled intracranial aneurysms: influence of aneurysm size. Am J Neuroradiol. 2007;28:628‐634. 17416811 PMC7977342

[3] Liauw L , van Buchem MA , Spilt A , et al. MR angiography of the intracranial venous system. Radiology. 2000;214:678‐682. 10715029 10.1148/radiology.214.3.r00mr41678

[4] Saloner D . The AAPM/RSNA physics tutorial for residents. An introduction to MR angiography. Radiographics. 1995;15:453‐465. 7761648 10.1148/radiographics.15.2.7761648

[5] Qiao Y , Steinman DA , Qin Q , et al. Intracranial arterial wall imaging using three‐dimensional high isotropic resolution black blood MRI at 3.0 Tesla. J Magn Reson Imaging. 2011;34:22‐30. 21698704 10.1002/jmri.22592

[6] Parker DL , Yuan C , Blatter DD . MR angiography by multiple thin slab 3D acquisition. Magn Reson Med. 1991;17:434‐451. 2062215 10.1002/mrm.1910170215

[7] Meixner CR , Liebig P , Speier P , et al. High resolution time‐of‐flight MR‐angiography at 7 T exploiting VERSE saturation, compressed sensing and segmentation. Magn Reson Imaging. 2019;63:193‐204. 31434005 10.1016/j.mri.2019.08.014

[8] Kopeinigg D , Aksoy M , Forman C , et al. Prospective optical motion correction for 3D time‐of‐flight angiography. Magn Reson Med. 2013;69:1623‐1633. 22887025 10.1002/mrm.24423

[9] Griswold MA , Jakob PM , Heidemann RM , et al. Generalized autocalibrating partially parallel acquisitions (GRAPPA). Magn Reson Med. 2002;47:1202‐1210. 12111967 10.1002/mrm.10171

[10] Pruessmann KP , Weiger M , Scheidegger MB , Boesiger P . SENSE: sensitivity encoding for fast MRI. Magn Reson Med. 1999;42:952‐962. 10542355

[11] Lustig M , Donoho DL , Santos JM , Pauly JM . Compressed sensing MRI. IEEE Signal Process Mag. 2008;25:72‐82.

[12] Yamamoto T , Fujimoto K , Okada T , et al. Time‐of‐flight magnetic resonance angiography with sparse undersampling and iterative reconstruction: comparison with conventional parallel imaging for accelerated imaging. Invest Radiol. 2016;51:372‐378. 26561046 10.1097/RLI.0000000000000221

[13] Yamamoto T , Okada T , Fushimi Y , et al. Magnetic resonance angiography with compressed sensing: an evaluation of moyamoya disease. PloS One. 2018;13:e0189493. 29351284 10.1371/journal.pone.0189493 PMC5774704

[14] Lin Z , Zhang X , Guo L , et al. Clinical feasibility study of 3D intracranial magnetic resonance angiography using compressed sensing. J Magn Reson Imaging. 2019;50:1843‐1851. 30980468 10.1002/jmri.26752

[15] Sandino CM , Cheng JY , Chen F , Mardani M , Pauly JM , Vasanawala SS . Compressed sensing: from research to clinical practice with deep neural networks: shortening scan times for magnetic resonance imaging. IEEE Signal Process Mag. 2020;37:117‐127. 10.1109/MSP.2019.2950433 PMC7664163 33192036

[16] Bilgic B , Gagoski BA , Cauley SF , et al. Wave‐CAIPI for highly accelerated 3D imaging. Magn Reson Med. 2015;73:2152‐2162. 24986223 10.1002/mrm.25347 PMC4281518

[17] Gagoski BA , Bilgic B , Eichner C , et al. RARE/turbo spin echo imaging with simultaneous multislice wave‐CAIPI. Magn Reson Med. 2015;73:929‐938. 25640187 10.1002/mrm.25615 PMC4334698

[18] Polak D , Setsompop K , Cauley SF , et al. Wave‐CAIPI for highly accelerated MP‐RAGE imaging. Magn Reson Med. 2018;79:401‐406. 28220617 10.1002/mrm.26649 PMC5563495

[19] Polak D , Cauley S , Huang SY , et al. Highly‐accelerated volumetric brain examination using optimized wave‐CAIPI encoding. J Magn Reson Imaging. 2019;50:961‐974. 30734388 10.1002/jmri.26678 PMC6687581

[20] Richter JA , Wech T , Weng AM , et al. Free‐breathing self‐gated 4D lung MRI using wave‐CAIPI. Magn Reson Med. 2020;84:3223‐3233. 32767457 10.1002/mrm.28383

[21] Longo M , Conklin J , Cauley S , et al. Evaluation of ultrafast wave‐CAIPI MPRAGE for visual grading and automated measurement of brain tissue volume. Am J Neuroradiol. 2020;41:1388‐1396. 32732274 10.3174/ajnr.A6703 PMC7658899

[22] Richter JA , Wech T , Weng AM , et al. Accelerated aortic 4D flow MRI with wave‐CAIPI. Magn Reson Med. 2021;85:2595‐2607. 33231886 10.1002/mrm.28605

[23] Chen F , Zhang T , Cheng JY , Shi X , Pauly JM , Vasanawala SS . Autocalibrating motion‐corrected wave‐encoding for highly accelerated free‐breathing abdominal MRI. Magn Reson Med. 2017;78:1757‐1766. 27943402 10.1002/mrm.26567 PMC5466545

[24] Cho J , Liao C , Tian Q , et al. Highly accelerated EPI with wave encoding and multi‐shot simultaneous multislice imaging. Magn Reson Med. 2022;88:1180‐1197. 35678236 10.1002/mrm.29291

[25] Breuer FA , Blaimer M , Mueller MF , et al. Controlled aliasing in volumetric parallel imaging (2D CAIPIRINHA). Magn Reson Med. 2006;55:549‐556. 16408271 10.1002/mrm.20787

[26] Su S , Qiu Z , Luo C , et al. Accelerated 3D bSSFP using a modified wave‐CAIPI technique with truncated wave gradients. IEEE Trans Med Imaging. 2020;40:48‐58. 32886608 10.1109/TMI.2020.3021737

[27] Wang H , Qiu Z , Su S , et al. Parameter optimization framework on wave gradients of wave‐CAIPI imaging. Magn Reson Med. 2020;83:1659‐1672. 31658397 10.1002/mrm.28034

[28] Uecker M , Lai P , Murphy MJ , et al. ESPIRiT—an eigenvalue approach to autocalibrating parallel MRI: where SENSE meets GRAPPA. Magn Reson Med. 2014;71:990‐1001. 23649942 10.1002/mrm.24751 PMC4142121

[29] Duyn JH , Yang Y , Frank JA , van der Veen JW . Simple correction method for k‐space trajectory deviations in MRI. J Magn Reson. 1998;132:150‐153. 9615415 10.1006/jmre.1998.1396

[30] Uecker M , Ong F , Tamir JI , et al. Berkeley advanced reconstruction toolbox. In Proceedings of the 23rd Annual Meeting of ISMRM, Toronto, Ontario, Canada; 2015:2486.

[31] Lustig M , Donoho D , Pauly JM . Sparse MRI: the application of compressed sensing for rapid MR imaging. Magn Reson Med. 2007;58:1182‐1195. 17969013 10.1002/mrm.21391

[32] Bilgic B , Ye H , Wald LL , Setsompop K . Simultaneous time interleaved multislice (STIMS) for rapid susceptibility weighted acquisition. Neuroimage. 2017;155:577‐586. 28435102 10.1016/j.neuroimage.2017.04.036 PMC5511575

[33] Robson PM , Grant AK , Madhuranthakam AJ , Lattanzi R , Sodickson DK , McKenzie CA . Comprehensive quantification of signal‐to‐noise ratio and g‐factor for image‐based and k‐space‐based parallel imaging reconstructions. Magn Reson Med. 2008;60:895‐907. 18816810 10.1002/mrm.21728 PMC2838249

[34] de Buck MHS , Jezzard P , Hess AT . Optimization of undersampling parameters for 3D intracranial compressed sensing MR angiography at 7 T. Magn Reson Med. 2022;88:880‐889. 35344622 10.1002/mrm.29236 PMC9314035

[35] Mitchell DG , Tasciyan T , Ortega HV , Outwater E , Vinitski S . Pulsation artifact in short TR MR imaging and angiography: exacerbation with signal averaging. J Magn Reson Imaging. 1994;4:709‐718. 7981516 10.1002/jmri.1880040514

[36] Frank LR , Buxton RB , Kerber CW . Pulsatile flow artifacts in 3D magnetic resonance imaging. Magn Reson Med. 1993;30:296‐304. 8412600 10.1002/mrm.1910300305

[37] Drangova M , Pelc NJ . Artifacts and signal loss due to flow in the presence of Bo inhomogeneity. Magn Reson Med. 1996;35:126‐130. 8771030 10.1002/mrm.1910350116

[38] Lustig M , Pauly JM . SPIRiT: iterative self‐consistent parallel imaging reconstruction from arbitrary k‐space. Magn Reson Med. 2010;64:457‐471. 20665790 10.1002/mrm.22428 PMC2925465

[39] Kim TH , Bilgic B , Polak D , Setsompop K , Haldar JP . Wave‐LORAKS: combining wave encoding with structured low‐rank matrix modeling for more highly accelerated 3D imaging. Magn Reson Med. 2019;81:1620‐1633. 30252157 10.1002/mrm.27511 PMC6347537

[40] Cauley SF , Setsompop K , Bilgic B , Bhat H , Gagoski B , Wald LL . Autocalibrated wave‐CAIPI reconstruction; joint optimization of k‐space trajectory and parallel imaging reconstruction. Magn Reson Med. 2017;78:1093‐1099. 27770457 10.1002/mrm.26499 PMC5400736

[41] Zhang T , Pauly JM , Vasanawala SS , Lustig M . Coil compression for accelerated imaging with Cartesian sampling. Magn Reson Med. 2013;69:571‐582. 22488589 10.1002/mrm.24267 PMC3396763

[42] Hansen MS , Sørensen TS . Gadgetron: an open source framework for medical image reconstruction. Magn Reson Med. 2013;69:1768‐1776. 22791598 10.1002/mrm.24389

[43] Ye JC . Compressed sensing MRI: a review from signal processing perspective. BMC Biomed Eng. 2019;1:1‐17. 32903346 10.1186/s42490-019-0006-z PMC7412677

[44] Chen C , Huang J . Exploiting the wavelet structure in compressed sensing MRI. Magn Reson Imaging. 2014;32:1377‐1389. 25153483 10.1016/j.mri.2014.07.016

