# Supplementary material for: Highly accelerated intracranial time‐of‐flight magnetic resonance angiography using wave‐encoding
Source: Magn Reson Med. 2023 Apr 3;90(2):432–43. doi: 10.1002/mrm.29647 (PMC10953028; doi:10.1002/mrm.29647)
Supplement: Supplementary file 1 — FIGURE S1. Comparison of retrospectively undersampled reconstructions using the 2D‐CAIPI sampling scheme at different acceleration factors for a synthesized Cartesian dataset derived from a fully sampled wave‐encoded dataset and a reference Cartesian dataset acquired using the conventional method. FIGURE S2. Comparison of retrospectively undersampled reconstructions using the CS sampling scheme at different acceleration factors for a synthesized Cartesian dataset derived from a fully sampled wave‐encoded dataset and a reference Cartesian dataset acquired using the conventional method. TABLE S1. Results from the quantitative analyses of the CBR between vessels and static background tissue at different acceleration factors, SSIM and Vessel‐masked SSIM between the MIP images obtained from the undersampled and fully sampled reconstructions (N = 6). [file MRM-90-432-s001.docx]

**Supporting Information**

|  |  | **wave-CAIPI** | **2D-CAIPI** | **wave-CS** | **CS** |
| --- | --- | --- | --- | --- | --- |
| **R4** | **CBR** | 6.65 ± 0.0 | 6.48 ± 0.09 | 6.30 ± 0.07 | 6.11 ± 0.06 |
|  | **SSIM** | 0.923 ± 0.016 | 0.885 ± 0.019 | 0.845 ± 0.022 | 0.823 ± 0.020 |
|  | **vessel-masked SSIM** | 0.993 ± 0.001 | 0.989 ± 0.001 | 0.980 ± 0.001 | 0.969 ± 0.001 |
| **R6** | **CBR** | 6.34 ± 0.05 | 5.25 ± 0.15 | 6.14 ± 0.05 | 5.82 ± 0.10 |
|  | **SSIM** | 0.864 ± 0.024 | 0.653 ± 0.027 | 0.816 ± 0.028 | 0.756 ± 0.027 |
|  | **vessel-masked SSIM** | 0.985 ± 0.002 | 0.932 ± 0.006 | 0.975 ± 0.002 | 0.954 ± 0.002 |
| **R8** | **CBR** | 5.97 ± 0.04 | 4.15 ± 0.20 | 5.83 ± 0.03 | 5.12 ± 0.20 |
|  | **SSIM** | 0.795 ± 0.031 | 0.439 ± 0.024 | 0.758 ± 0.033 | 0.667 ± 0.026 |
|  | **vessel-masked SSIM** | 0.971 ± 0.004 | 0.810 ± 0.015 | 0.961 ± 0.005 | 0.921 ± 0.006 |

**Table S1**: Results from the quantitative analyses of the CBR between vessels and static tissue background at different acceleration factors, SSIM and Vessel-masked SSIM between the MIP images obtained from the undersampled and fully sampled reconstructions (N=6).


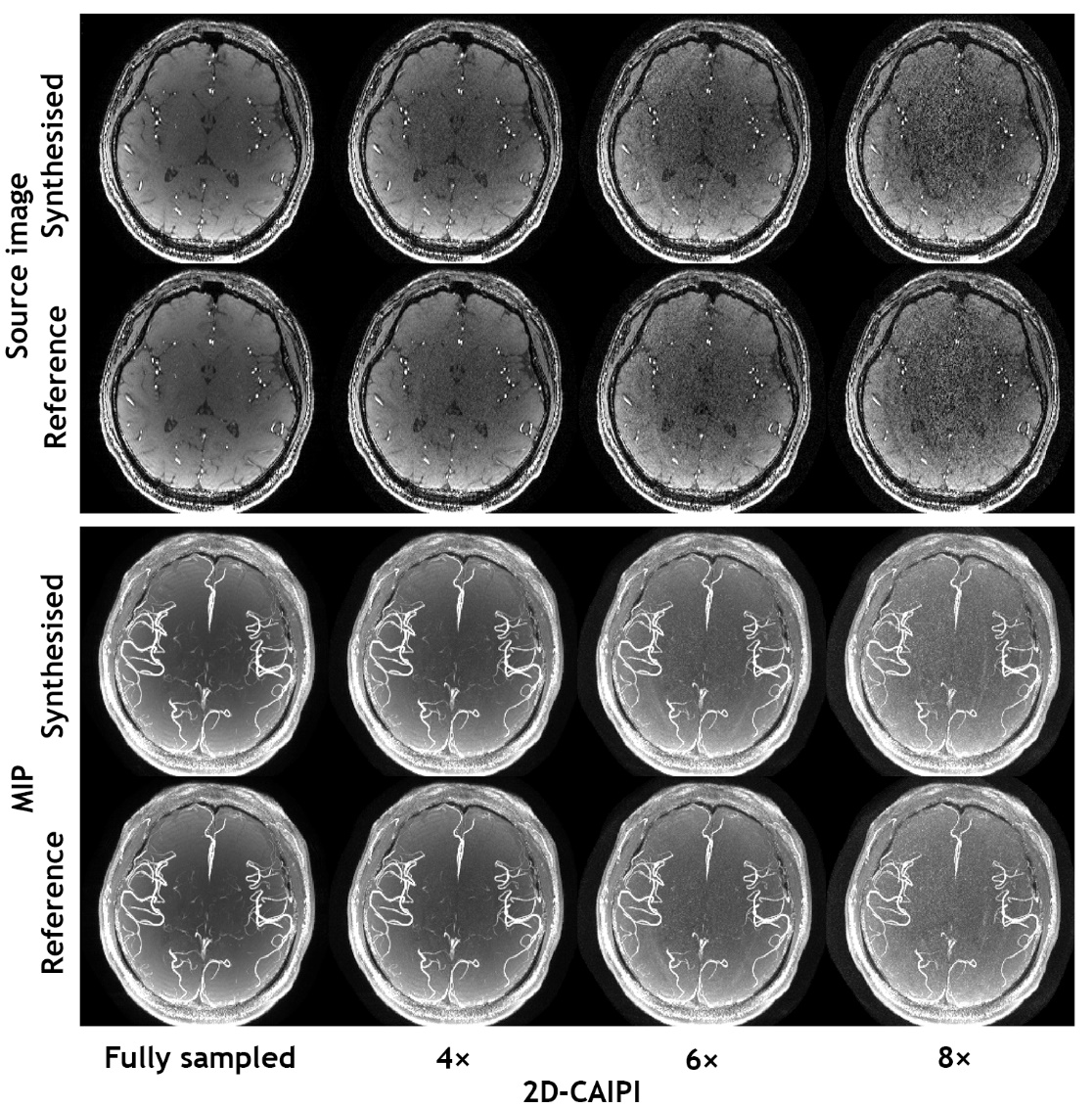


**Figure S1.** Comparison of retrospectively undersampled reconstructions using the 2D-CAIPI sampling scheme at different acceleration factors for a synthesized Cartesian dataset derived from a fully sampled wave-encoded dataset and a reference Cartesian dataset acquired using the conventional method.


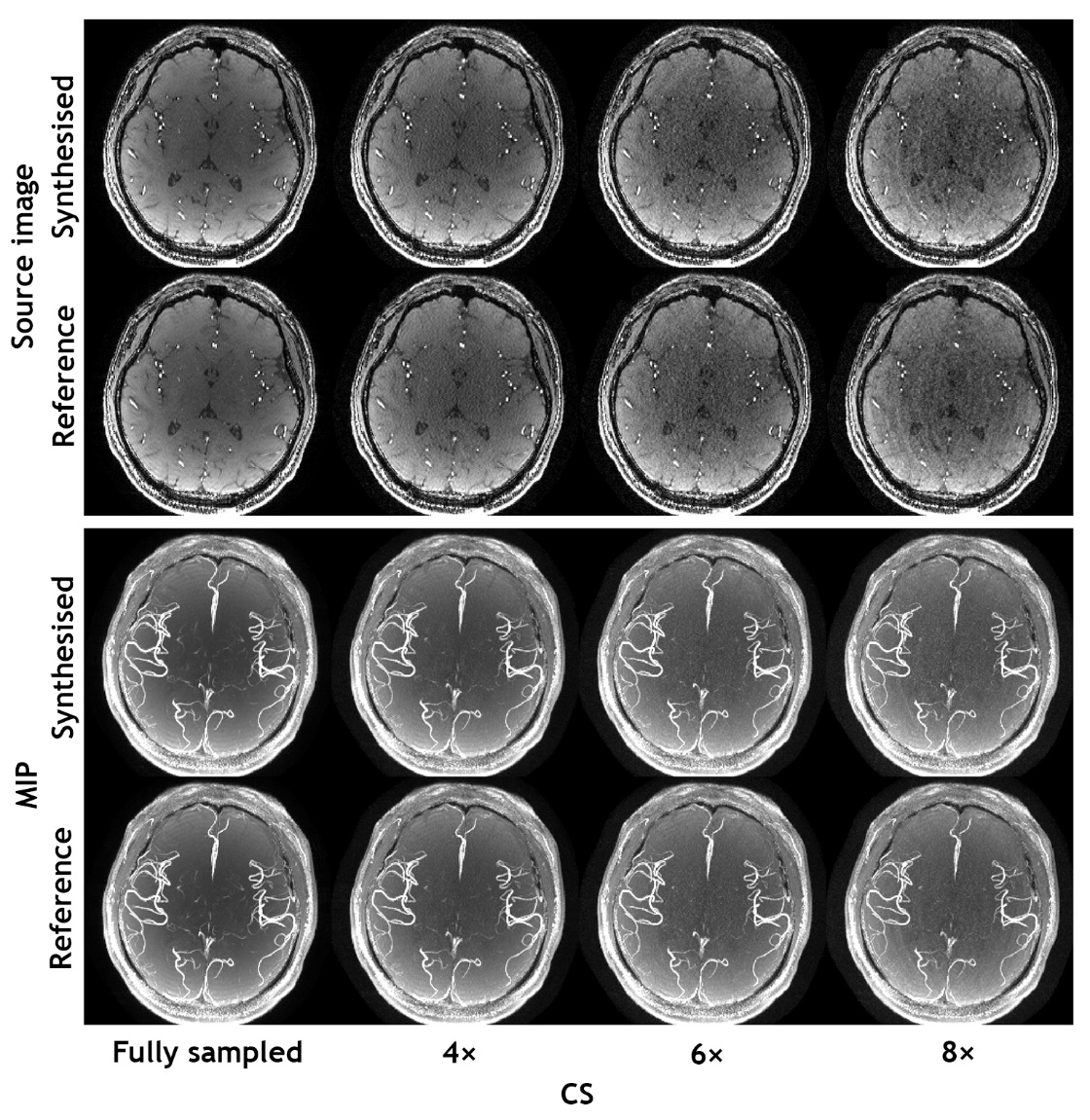


**Figure S2.** Comparison of retrospectively undersampled reconstructions using the CS sampling scheme at different acceleration factors for a synthesized Cartesian dataset derived from a fully sampled wave-encoded dataset and a reference Cartesian dataset acquired using the conventional method.
